# Selected abstracts from the 2019 Simulation Summit: Winnipeg, Canada. 07-08 November 2019

**DOI:** 10.1186/s41077-019-0110-0

**Published:** 2019-09-19

## 2019 Simulation Summit/Sommet de simulation 2019

### 02 Material concepts: Integrating theory and practice during simulation-based training to support procedural skills retention and transfer

#### J. J.H. Cheung^1^, K. M. Kulasegaram^2^, N. N. Woods^2^, R. Brydges^3^

##### ^1^University of Toronto, Toronto, ON, Canada; ^2^The Wilson Centre, Toronto, ON, Canada; ^3^St. Michael's Hospital & University of Toronto, Toronto, ON, Canada

###### **Correspondence:** J. J.H. Cheung

**Background:** Instruction that encourages trainees to integrate conceptual “why” and procedural “how” knowledge improves their transfer of procedural skills. For training away from the bedside and direct supervision, questions remain on how to represent the causal relationship between clinical concepts and procedural actions (e.g., how patient anatomy relates to inserting a needle). Simulation presents a unique education modality for delivering causal instruction that can help trainees build cognitive connections between the theoretical concepts and procedural actions of clinical skills. We varied the modality and level of interactivity when presenting these causal relationships during simulation-based lumbar puncture (LP) training and measured impacts on participants’ retention and transfer.

**Research Question:** When delivering integrated instruction, how does the modality (video-based or simulation-based) impact trainees’ skill retention and transfer?

**Methods:** During a 1-hour session, we randomized 66 medical students to one of three instructional interventions: i) procedural-only video-based instruction, ii) integrated video-based instruction, and iii) integrated simulation-based instruction. One-week later, we tested participants’ LP skill retention and transfer, and their conceptual knowledge on a written test.

**Results:** Simple mediation regression analyses revealed that participants receiving integrated instruction had superior LP retention and transfer skills via gains in conceptual knowledge (all *p*<0.01). We found no significant performance differences between the integrated groups (*p*>0.01). Participants receiving procedural-only instruction practiced significantly more LPs during training (*M*=2.36) than participants receiving integrated video-based (*M*=1.82) and simulation-based instruction (*M*=1.50), *p*<0.05.

**Conclusion:** Trainees’ ability to create cognitive connections between conceptual and procedural knowledge appears to improve when they interact with instructional materials highlighting the causal relationships between these knowledge types. Simulation experiences can be designed to make abstract clinical concepts visible using hands-on, interactive modules, which enhances trainees’ conceptual knowledge, as well as, their skill retention and transfer. However, integrated instruction reduced participants’ time to practice LP scenarios, which may have reduced the effectiveness of our efforts to promote such “cognitive integration”. We suggest that more advanced trainees with baseline procedural proficiency may experience greater benefits from such integrated instruction. Integrated instruction may improve trainees’ skill retention and transfer, despite reducing how many LPs they could practice.

### 03 Does utilization of an intubation safety checklist reduce dangerous omissions during simulated resuscitation scenarios?

#### C. Forristal^1^, S. Mal^1^, M. Columbus^1^, K. Van Aarsen^1^, D. Ouellette^1^, N. Farooki^2^, K. Hayman^2^, N. Smith^3^, S. McLeod^4^

##### ^1^Western University, London, ON, Canada; ^2^University of Toronto, Toronto, ON, Canada; ^3^London Health Sciences Centre, London, ON, Canada; ^4^Scwartz/Reisman Emergency Medicine Institute, Toronto, ON, Canada

###### **Correspondence:** C. Forristal

**Introduction:** Airway management is a high-risk procedure regularly performed by emergency medicine (EM) physicians. Studies demonstrate an increase in adverse events associated with airway management outside the operating theatre, with errors of omission being the most common type of human error. To address this risk, checklists are becoming a common pre-intubation tool. Simulation is a safe setting in which to study the implementation of a new airway checklist. The purpose of this study was to determine if a novel airway checklist decreases the rate of important task omission during simulated resuscitation scenarios.

**Methods:** This was a dual-centre, randomized controlled trial of a novel airway checklist utilized by EM practitioners in a simulated environment. The 29-item peri-intubation checklist was derived by experienced EM practitioners following a review of published airway checklists. Participants were EM residents or physicians who work more than 20 hours/month in an emergency department. Volunteers were recruited from 2 academic centres to complete 3 simulated scenarios (2 requiring intubation, 1 cricothyroidotomy), and were randomized to standard care or checklist use. A minimum of 2 assessors documented the number of omitted tasks deemed important in airway management and the time until definitive airway management. Discrepancies between assessors were resolved by single-assessor video review.

**Results:** Fifty-four EM practitioners participated. Baseline characteristics were not significantly different between the study groups. The average percentage of omitted tasks over 3 scenarios was 45.7% in the control group (n=25) and 13.5% in the checklist group (n=29) – an absolute difference of 32.2% (95% CI: 27.8%, 36.6%). Time to intubation was significantly longer in the checklist group for the first 2 scenarios (mean difference 114.10s, 95% CI: 48.21s, 179.98s and 76.34s 95% CI: 31.35s, 121.33s), but not significantly different in the third scenario where cricothyroidotomy was required (mean difference 33.75s, 95% CI: -28.14s, 95.65s).

**Conclusion:** In a simulated setting, use of an airway checklist significantly decreased the omission rate of important airway management tasks; however it increased the time to definitive airway management. Further study is required to determine if these findings are consistent in a clinical setting and how they impact the rate of adverse events.

### 04 Teaching skills via kinesthetic sensory pathway

#### B. Zheng

##### University of Alberta, Edmonton, AB, Canada

**Introduction:** When learning a particular motor skill, we often acquire information through visual and kinesthetic sensory pathways. We extensively study the role of visual feedback on skill acquisition, but our knowledge on kinesthetic feedback remains rudimentary. In this study, we examined the role of kinesthetic feedback in learning laparoscopic skills. It was our hypothesis that this kinesthetic guidance received from the expert would facilitate skill learning. This was carried out as part of results from a larger project.

**Methods:** At the Surgical Simulation Research Lab of the University of Alberta, we implemented an advance master-slave training system connecting 4 SensAble™ PHANTOM^®^ Omni that enabled us to record an expert surgeons’ kinesthetic features from both hands and deliver those features to a surgeon-in-training while practicing a laparoscopic skill. Twelve novice medical students were asked to perform a pattern-cutting task within a laparoscopic training box. Each subject was required to perform 30 minutes per day over a six-day training phase. Six subjects in the *kinesthetic-guided* training group had the additional utilization of a device that allowed movement to be directly transmitted from one operator to the other, thereby allowing them to feel exactly what an expert would do under the same task. Another six subjects in the *self-directed* learning group received no such a feedback. Precision cutting was chosen as the surgical task due to its fidelity to real surgery and ease of analysis. Performance was evaluated by the time to completion and precision of the cut. Error analysis was completed via computer analysis of average deviations from the original line. The scores were averaged between the individuals of each group for each session and plotted over 6 sessions to yield a learning curve. Task time and cutting error duration were reduced over training phase.

**Results:** The skill acquisition was significantly faster in the kinesthetic-guided than the self-directed training group, with decreasing amount of performance errors.

**Discussion:** Kinesthetic feedback helps trainees to build surgical skills. Further work on correlating error analysis to video data could reveal more data regarding where and when to use haptic feedback as a future strategy to augment current and traditional surgical learning.

### 05 The nature of learning from simulation

#### F. Shariff, G. Regehr, R. Hatala

##### University of British Columbia, Vancouver, BC, Canada

###### **Correspondence:** F. Shariff

**Background:** Ongoing learning in complex clinical environments requires health professionals to assess their own performance, manage their learning, and modify their practices based on self-monitored progress. Self-regulated learning (SRL) theory suggests that while learners may be capable of such learning, they often need guidance to enact it effectively. Debriefings in simulation may be an ideal time to prepare learners for self-regulated learning in targeted areas, but may not be optimally fostering these practices. The goal of this study was to explore and characterize the nature of the learning by participants after team-based simulation training.

**Methods:** A qualitative study informed by grounded theory methodology was conducted in the context of three Interprofessional in-situ trauma simulations at our level 1 trauma center. 18 participants were interviewed immediately, and in follow-up 4-6 weeks after the simulation experience. Transcripts were analyzed in an iterative constant comparative approach to explore emergent concepts and themes surrounding our research question.

There were many examples of acquired content knowledge and straightforward practice change plans during initial interviews; however, more sophisticated examples of self-regulated learning were lacking early on. Some participants appeared to have evolved more specific learning goals and rudimentary plans for self-regulated implementation and improvement by the follow up interviews, but often suggested this was prompted by the study interview questions rather than the simulation debriefing itself.

**Conclusion:** Overall, participants did not engage in fulsome development of SRL plans based on the simulation and debriefing; however, there were elements of SRL present, particularly in follow-up discussions after participants were given time to reflect on the interview questions and their own goals. This is an encouraging sign that simulation training can support development of this skill. However, debriefing approaches would need to be better optimized to take full advantage of the opportunity to encourage and foster SRL in practice after the simulation is over.

### 07 Simulation to assess teaching competence: Validity evidence

#### L. Rodriguez Guerineau, B. Mema

##### The Hospital for Sick Children, Toronto, ON, Canada

###### **Correspondence:** L. Rodriguez Guerineau

**Background:** Teaching competence is expected for all trainees, especially the ones that will be working in teaching centers (academic institutions). Direct training in teaching competencies is scarce, apprenticeship-like when it occurs, and while trainees are involved in teaching other trainees, this type of activity is largely unsupervised and not assessed by expert teachers for many reasons (intermittent nature, time-constraint, expertise of assessor). Simulation is attractive for this type of assessment as (1) it satisfies both the structural and functional fidelity of a teaching encounter (2) it permits assessment by an expert, and (3) scenarios can be condensed to encompass many of the essential teaching competencies. The purpose of this study was to collect validity evidence for using a simulation program to train and assess clinician teachers.

**Methods:** We used Kane’s framework to collect validity evidence. The purpose of the assessment was formative and the creation of the blueprint of the simulation scenarios was based on an article (Literature review and expert consensus: “Teaching as a Competency”: competencies for medical educators. Acad Med. 2011; 86(10):1211-20), and on the teaching Entrustable Professional Activity for our specialty.

The assessment tools for each of the stations were built using literature reviews and, when necessary, interviews with different stakeholders such as: patient, trainee, healthcare team and educators. Participant trainees were debriefed immediately after each scenario ended.

**Results:** To assess all competencies we designed 3 simulation stations: Bedside teaching, Managing a complex clinical situation while teaching a trainee, and Feedback. Assessment tools in a form of Global Rating Scale (GRS) and allowing qualitative remarks were piloted. Raters were expert educators, learners and other health-professions educators. The raters reported ease of using the assessment tools and that the tools fully captured the qualities of a good teacher.

**Conclusions:** Teacher training and assessment is often scarce and largely unsupervised. We collected evidence for purpose and scoring, using a 3-station simulation program. Simulation is an attractive tool to train the teacher as it satisfies the fidelity of a teaching encounter, and allows for assessment and feedback by an expert.

### 09 Rethinking Continuous Professional Development (CPD): Building a simulation program through collaboration

#### M. Tremblay, S. Daniel, M. Bouchard, P. Wade

##### Fédération des médecins spécialistes du Québec, Montréal, QC, Canada

###### **Correspondence:** M. Tremblay

**Background:** The Maintenance of Certification (MOC) program requires specialists to complete 25 self-assessment credits over 5 years. New regulation in Quebec requires 10 hours of practice assessment, including simulation.

**Methods:** After a comprehensive needs assessment, we concluded that centers focused the majority of their programs on students and simulation programs for specialists were limited. Course description and learning objectives were difficult to find on organizations’ websites. To meet our members’ needs, we collaborated with five centers to update their courses and several experts to develop three new sessions for specialists. The objective was to develop long-term partnerships with accredited simulation centers and experts. To offer multiple simulation sessions in a one-day program, we added a second day to our annual congress.

**Results:** In 2017, our one-day program offered 13 simulations in 5 centers. 250 medical specialists experimented with simulation as a new CPD approach. Participants were highly satisfied with the content and the experts. 90% of participants stated they would change specific aspects of their practice. 95% stated they would participate in simulation again. Using standardize assessment tools, experts from every session reported an increase in participants’ competencies. We encountered skepticism from Program Directors as to the need for simulation programs for specialists. Developing programs tailored to their needs was a challenge. Support from our Board of Directors and our perseverance ensured success.

**Conclusion:** Rethinking CPD needs for physicians and developing partnerships allows organizations to offer innovative programs. We are measuring long-term impact of simulation sessions from 2017. In 2018, we offered our 2^nd^ simulation day and added new partners.

### 10 The impact of a crisis resource management simulation outreach program on the development of collective-efficacy in interprofessional teams in rural hospitals throughout Northern Ontario

#### M. Roach^1^, N. Lafreniere^2^, R. Anderson^3^

##### ^1^Health Sciences North, Sudbury, ON, Canada; ^2^Laurentian University, Sudbury, ON, Canada; ^3^Northern Ontario School of Medicine, Sudbury, ON, Canada

###### **Correspondence:** M. Roach

**Introduction:** In emergency departments there is the potential for high risk, low occurrence crises to occur at any time. Good patient outcomes often hinge on effective interprofessional teamwork. Nowhere is this more evident than in rural and remote hospitals, where resources are short and assistance can be far away.

**Methods:** Poor patient outcomes during a crisis are often not a result of an individual person’s clinical knowledge or skill but due to a combination of non-technical skills involving the entire interprofessional team. Crisis resource management (CRM) is a concept that encompasses the non-technical skills used by a team during a crisis. Proper integration of CRM by interprofessional teams during a crisis leads to improved patient outcomes. Although the use of simulation to teach CRM is common, many rural community hospitals do not have access to simulation locally often requiring individual team members to travel to larger centres for training. This prevents team members from training together, in their environment using their equipment. Individuals training apart from their teams and environment can decrease the transferability of skills into practice; reduce team cohesion and development of collective-efficacy. Collective-efficacy is a group’s shared belief in its ability to perform behaviours collectively and directly influences the quality of a team’s performance, leading to successful outcomes.

**Results:** Simulation can increase the collective-efficacy of interprofessional teams, but this is only possible if teams are trained together. Health Sciences North has developed a simulation outreach program that provides rural facilities throughout Northern Ontario with access to high quality interprofessional team based simulation, improving team performance and patient outcomes.

**Conclusion:** The purpose of this study is to examine if a mobile simulation program impacts the development of collective-efficacy in interprofessional teams working in rural emergency departments throughout the NELIHN and examine the transfer of the learned CRM skills and behaviours into the clinical environment. The results of this study will be used to assist in increasing accessibility of high quality simulation throughout Northern Ontario, standardizing CRM practice throughout the region and developing a program evaluation model that can accurately measure the impact of in-situ simulation training on the collective-efficacy of interprofessional teams.

### 11 Simulation in the continuing professional development of Canadian academic emergency physicians – a national survey

#### C. Forristal^1^, K. Woolfrey^1^, A. Hall^2^, E. Russell^2^, A. Szulewski^2^, T. Chaplin^2^, T. McColl^3^, A. Petrosoniak^4^, G. N. Mastoras^5^, K. Caners^6^, B. Thoma^7^, J. Lewis. Huffman^9^, C. Dakin^10^

##### ^1^Western University, London, ON, Canada; ^2^Queen's University, Kingston, ON, Canada; ^3^University of Manitoba, Winnipeg, MB, Canada; ^4^St. Michael's Hospital, Toronto, ON, Canada; ^5^The Ottawa Hospital, Ottawa, ON, Canada; ^6^McMaster University, Burlington, ON, Canada; ^7^University of Saskatchewan, Saskatoon, SK, Canada; ^9^University of Calgary, Calgary, AB, Canada; ^10^Vancouver General Hospital, Vancouver, BC, Canada

###### **Correspondence:** C. Forristal

**Background:** Capitalizing on the success of Simulation-Based Education (SBE) in residency-training programs, simulation has been gradually integrated into Continued Professional Development (CPD) programs for Emergency Physicians (EPs) in Canada. This study sought to characterize how Canadian academic emergency medicine (EM) departments have implemented SBE for CPD.

**Methods:** Two national surveys were conducted. First, the National Faculty Simulation Status Assessment Survey was administered by telephone to the simulation directors (or equivalent) at 20 Canadian academic EM sites. Second, the Faculty Simulation Needs Assessment Survey was administered online to all full-time EPs across 9 Canadian academic EM sites.

**Results:** The response rates for the National Status and Needs Assessment Surveys were 100% (20/20), and 40% (252/635), respectively. The majority (60%) of Canadian academic EM sites reported utilizing SBE for CPD, though only 30% reported dedicated funding support. The median EP reported annual amount of SBE was 3 hours (IQR 1-6 hours). Reported incentivization offered in the form of continued medical education credits varied between simulation directors (67%) and EPs (44%). The top barriers to using SBE identified by simulation directors were: lack of faculty time, fear of peer judgment, and faculty inexperience. In contrast, EPs identified time commitments outside of shift, lack of opportunities, and lack of departmental funding as their top barriers. The top three EP SBE content areas of interest were: performance of rare procedures, pediatric resuscitation, and neonatal resuscitation. Interprofessional involvement in SBE CPD was valued by both simulation directors and EPs, with most EPs (79%) indicating it is useful.

**Conclusions:** Most Canadian EPs and simulation directors recognize the value of SBE for CPD, yet it is utilized infrequently by only two-thirds of Canadian academic EM departments. Further, SBE for CPD is perceived as being inadequately incentivized. Discrepancies in responses between simulation directors and EPs with respect to barriers to SBE for CPD suggests the need for dialogue about how best proceed with program implementation. As SBE for CPD is incorporated more frequently, and at more sites, desired EP identified content should be a focus (e.g. rare procedures), and interprofessional involvement should be encouraged.

### 13 Multi-source feedback during simulated resuscitation scenarios: A qualitative analysis

#### H. Braund^1^, T. Chaplin^1^, A. Szulewski^1^, N. Dalgarno^1^, R. Egan^1^, B. Thoma^2^

##### ^1^Queen's University, Kingston, ON, Canada; ^2^University of Saskatchewan, Saskatoon, SK, Canada

###### **Correspondence:** H. Braund

**Background:** The implementation of competency-based medical education requires increased feedback based on direct observation, but this can be challenging for rare events such as resuscitation cases. Simulation provides an environment where such rare events can be practiced and observed.

**Objective:** We sought to explore the qualitative differences between assessments of residents provided by nurses,

co-residents, and attending physicians within a simulation-based training course focused on resuscitation.

**Methods:** The simulation-based course consisted of 12 resuscitation cases and was completed by 87 first year residents from 14 specialties at two Canadian institutions. Faculty, co-resident participants, and a nurse completed narrative assessments after each case. All qualitative comments were analyzed using an emergent thematic approach through open coding using NVivo software.

**Results:** Residents’ communication skills were frequently discussed across faculty, peers, and nurses. Faculty provided positive comments on diagnostic actions and constructive feedback regarding administering medication and remembering dosages. Peers focused on providing positive and constructive comments on leadership skills including the ability to delegate tasks and the extent to which the leader accepted suggestions from team members. Nurses provided a mixture of feedback on initial assessment and interventions.

**Conclusion:** Our analyses demonstrate that assessors from differing backgrounds focus on different aspects of resident performance. This suggests that though alternative assessors such as co-residents and nurses are often present at rare events such as resuscitation cases, they do not provide the same feedback. Future work could investigate how multi-source feedback could be integrated to provide a holistic picture of resident performance.

### 14 Incorporating the patient’s voice into the debriefing: The opportunities and challenges of integrating patient partners, SPs, and clinicians

#### I. Cruz-Panesso, S. Gharavi, P. Drolet

##### Université de Montréal, Montréal, QC, Canada

###### **Correspondence:** I. Cruz-Panesso

**Background:** Despite the technological innovations in the field of healthcare simulation, there is only a paucity of work looking at how to best address the challenges posed by the debriefing. In this qualitative study, we explored the possibility of adding to the debriefing the perspective of two different groups of patients, the standardized patients (SPs) and the patient-as partners (PPs).

**Methods:** Through a focus group discussion (FGD), we addressed the various roles of SPs and PPs in providing feedback to medical students. During the FGD, SPs (*n* = 5), PPs (*n* = 1), faculty members and medical instructors (*n* = 5), and instructional designers (*n* = 2) talked about their motivation to be part of a simulation-based learning environment and how they viewed their roles, actual and potential, in the teaching/learning formula. The FGD was complemented with discussion meetings with administrative representatives from the patient-partnership office. Audio recordings and field notes from the FGD and all meetings were transcribed verbatim and analysed.

**Results:** Although SPs and PPs both emphasize the attitudinal aspects of simulation training, their different skill sets target varying components with regard to communication and empathy, leading to different types of feedback. SPs focus on the genuineness of the students’ interaction with the patients they played. They look at variables such as body language, tone of voice, and students’ emotional engagement in role-playing when assessing students’ social competency. On the other side, PPs have the expertise of living with a certain medical condition on a daily basis, which gives them a unique knowledge of the disease including what works and what does not when addressing their various problems. The PPs are more likely to observe and react to the students’ ability to communicate medical information at a level they can understand and with the appropriate level of compassion to make them accept their medical condition.

**Conclusion:** This study only begins to shed light on the nature and characteristics of separate and joint feedback delivered by SPs and PPs. The objectives and the approaches for continuing development opportunities in this area will be discussed.

### 15 Comparing the distensibility of 3D-printed thoracic aortic phantoms and normal human thoracic aortic tissue

#### M. Alomran

##### McGill University Health Centre, Montreal, QC, Canada

**Introduction:** Presently, aortic size is generally the most widely accepted parameter for elective intervention to prevent acute aortic syndrome, which continues to be associated with a significant burden of morbidity and mortality. However, it has been shown in the International registry of Acute Aortic Dissections that nearly 60% of aortic dissections occur in patients with thoracic aortas smaller than the cut-off size of 5.5 cm. This incentivized seeking more effective parameters to guide treatment; such as ultrasound derived biomechanics, including distensibility. In this experiment the ultrasound-derived distensibility of 3D-printed idealized thoracic aortic phantoms was compared with the previously acquired distensibility of human aortic tissue. This can eventually aid in validating these biomechanical parameters and the use of such phantoms in a simulation setting.

**Methods:** The aortic phantoms were manufactured out of a resin-like material using the Stratasys Object300 PolyJet 3D printer. The aortic phantoms were placed in a pressurized mock circuit to simulate the pressures that human aortas withstand. Diameters of the phantoms were obtained at multiple pressure points using ultrasound and was used to determine distensibility. The mean distensibility of 3 aortic phantoms was then compared to the mean distensibility of previously obtained 22 samples of healthy thoracic aortic tissue.

**Results:** The 3D-printed phantoms had a significantly higher mean distensibility than normal human tissue (3.87e^-03^ mmHg^-1^ vs 1.76e^-03^ mmHg^-1^, p = 0.0049).

**Conclusions:** Measurement of the biomechanical properties of 3D-printed thoracic aortic phantoms in a pressurized circuit is achievable and reproducible, which this experiment aimed to show as a proof of concept. The phantoms used in this experiment proved more distensible than normal human tissue due to the material used. However, this specific manufacturing method offer the possibility of printing multi-material models, can be used to manipulate directly-derived biomechanics. Hence, the models can be adjusted and tested continuously aiming for more human-like biomechanics. Eventually, models possessing human-like biomechanics can be used to validate novel ultrasound-derived biomechanical parameters and be utilized in simulation settings.

### 16 Evaluation of a simulation focused, three-dimensional printing network

#### A. Dubrowski^1^, R. Dubrowski^2^, N. S. Bishop^3^, G. Walsh^3^

##### ^1^Ontario Tech University, Oshawa, ON, Canada; ^2^Ontario Shores, Whitby, ON, Canada; ^3^Memorial University of Newfoundland, St. John's, NL, Canada

###### **Correspondence:** A. Dubrowski

**Background:** Simulation is ubiquitous in health professions education. However, in rural and remote settings, its utility is limited by costs and ease of access. To address these limitations, we implemented a three-dimensional (3D) design and printing network consisting of six rural sites and one central hub. Each site received a 3D printer, initial training, and continuing technical support from the central hub. The network aimed to provide rural and remote teaching hospitals with capabilities to produce innovative tools for simulation.

**Objective:** To evaluate the implementation process and success of the network.

**Methods:** Project leads at each of the six sites were contacted to participate in a survey and telephone interview. The survey, based on a utilization focused evaluation (UFE) framework, asked 31 short answer questions, organized in six categories: context, inputs, process, product, effectiveness, and sustainability. Telephone interviews were informed by stop, start, change, continue (SSCC) model. Data were analyzed using content analysis by two researchers.

**Results:** Five of the six sites participated. Survey results showed:

Context and inputs: The location of the 3D printer varied from medical office to hospital library. Users included physicians, nurses, and high school students.

Process: Most sites developed simulators in consultation with the hub. There was no site-to-site communication.

Products: Sites developed 1 to 10 products, including simulation tools and minor medical equipment.

Effectiveness: Sites found the design support insufficient at times.

Sustainability: Secure space and commitment from the central hub were identified as major contributors to sustainability.

Interviews showed similar patterns:

Stop: Nothing identified.

Start: Mechanism for site-to-site communication and more skills development opportunities.

Change: Increase network communication frequency.

Continue: Support from the central hub.

**Discussion:** Evaluation results support the initial concept of a simulation focused, 3D printing network to increase the use of simulation in rural and remote areas. Although the contextual set-up, resources and processes at each site varied, most sites were effective and expressed interest in continuing this work. Facilitators for future success and sustainability were: improved site-to-site collaborations and communication, and involvement of the central hub.

### 17 Getting them to the table: Engaging inter-professional team members to talk about their workplace practices

#### L. Nemoy, C. Léger, K. Daly, N. Khodadoust, D. Campbell, R. Brydges

##### St. Michael's Hospital, Toronto, ON, Canada

###### **Correspondence:** L. Nemoy

**Background:** Many researchers have used *in situ* simulation (ISS) successfully to observe real-time interactions and to identify latent safety threats (LSTs) in clinical settings. Yet ISS is resource-intensive and may not always work well as a stand-alone intervention, especially with clinicians with low exposure to simulation, where the expectation to perform in the ‘hot seat’, in front of colleagues can cause stress and reduce buy-in. As part of a quality improvement study, we combined multiple data collection techniques with an innovative approach to conducting tabletop simulations, wherein we combined ‘think-aloud’ and simulation principles to elicit healthcare professionals’ descriptions of how and why they act when providing collaborative care on a labour and delivery unit.

**Objective:** To reduce the concerns about the performative nature of ISS interventions, we developed tabletop simulation scenarios aimed at identifying the workplace practices and underlying rationalizations that intrapartum care providers engage when working through challenging clinical scenarios.

**Methods:** We engaged an interprofessional team of intrapartum clinicians, researchers, and simulation experts to help us design three tabletop simulation scenarios reflecting key interprofessional challenges, based on the data collected from incident analysis reports (n=81), field observations (n=17, 72 hours), and semi-structured interviews (n=15). With three separate teams of interprofessional policy-makers from the unit, we ran each scenario three times (n=9). We audio-recorded and transcribed the simulations and debriefs, and analyzed data using a ‘simple content analysis’.

**Results:** The tabletop simulations revealed ‘disjunctures’ in how different professionals interpreted and adhered to key policies and procedures. These findings supplemented our other data sources, enabling us to identify which issues to address in future simulation-based training, and which to address by other means. Moreover, participants described how our study opened a productive dialogue between professional groups and suggested the tabletop simulations might contribute to enhanced interprofessional understanding and cultural change.

**Conclusion:** We developed a process by which we can use tabletop simulations in different ways with slightly different populations, resulting in rich data, improved engagement, and greater potential for making informed change. This innovative tabletop simulation approach enabled us to reveal a crucial, unheard perspective that traditional ISS or centre-based simulation may not have yielded.

### 18 From sea to air: Surgical simulation in extreme environments

#### B. Eldreth^1^, A. J. LaPorta^1^, C. Brazell^1^, T. Hoang^1^, R. O. Rapada^1^, E. Pierce^2^, M. Pena^3^, M. Juliano^4^, R. Francoise^5^, N. Shattuck^6^, C. Bass^7^

##### ^1^Rocky Vista University, Parker, CO, United States; ^2^Naval Surface Warfare Center, Panama City, FL, United States; ^3^University of California, Davis, Davis, CA, United States; ^4^US Navy, Pensacola, United States; ^5^Vail Medical Center, Vail, United States; ^6^Naval Postgraduate School, Monterey, CA, United States; ^7^Duke University, Durham, NC, United States

###### **Correspondence:** B. Eldreth

**Background:** Advances in simulation have allowed us to test surgical tasks under adverse conditions. In this three-phase experiment, the U.S. Navy assessed the feasibility of placing modular resuscitation and operation rooms aboard small, mobile ships to reduce the time between battlefield injury and life-saving surgical care.

**Objective:** The purpose of this study is to quantify the ability of US Navy medical teams to perform critical surgical trauma resuscitation using high-fidelity patient simulators onboard US Navy ships under high sea states.

**Methods:** Three surgical teams were formed from 15 active duty military members. Simulated surgeries were performed aboard the USNS Brunswick using “Cut Suit” technology developed by Strategic Operations Inc to simulate four common battlefield injuries. Participants were fitted with individual monitors to assess dynamic and kinematic motion tracking and Motion Sickness Assessment Questionnaires (MSAQ) were given to participants. Each surgery was graded by subject matter experts on a Likert-type scale from zero to five with four or five deemed successful.

**Results:** 112 operations were performed in phase III and 89% were deemed successful with 46% completed at the roughest conditions tested. MSAQ scores revealed low motion sickness scores throughout most of the study and MSAQ positively correlated with sea state conditions (p<0.001). The only significant predictor for patient outcome scores was surgeon/team performing operations.

**Conclusions:** Surgical performance scores provide evidence that ship motion does not adversely affect surgical outcomes. Our next investigation tests the previous procedures in non-traditional aircraft such as the V-22, a vertical take-off and landing tiltrotor aircraft currently used by the U.S military for casualty evacuation. Phase IV will simulate the damage control tasks under V-22 motion conditions to further reduce the time between battlefield injury and surgical care.

### 19 Using simulation to educate caregivers: The dementia caregiver workshop

#### O. Monton^1^, I. Gélinas^1^, S. Gauthier^1^, L. Zahabi^1^, D. Weidner^1^, L. Simard-Villeneuve^1^, W. Claire^2^, F. Bhanji^3^, J. Morais^4^

##### ^1^McGill University, Montreal, QC, Canada; ^2^Caregiver Crosswalk Inc., Montreal, QC, Canada; ^3^Montréal Children's Hospital, Montréal, QC, Canada; ^4^McGill University Health Centre, Montréal, QC, Canada

###### **Correspondence:** O. Monton

**Background:** There are more than 747,000 Canadians living with dementia, with 25,000 new diagnoses every year. From the time of diagnosis, dementia caregivers are involved in every aspect of the disease and assume a number of roles and responsibilities throughout the illness trajectory. The increased demands placed on dementia caregivers have been shown to impact their physical, psychological, and emotional wellbeing.

Simulation is recognized for being a potent pedagogical tool with long lasting effects as it mimics real life situations. Our team has developed an interprofessional simulation-based workshop for dementia caregivers to address their needs.

**Objective:** The goal of this workshop is to educate caregivers about dementia and the progression of the disease with its demands, in an attempt to prepare them for the challenges and complexities of the caregiving experience.

**Methods:** This full-day interprofessional workshop includes: (1) a presentation on dementia and the behavioural and psychological symptoms of dementia, facilitated by a geriatrician or neurologist; (2) a discussion on navigating the health care system, facilitated by a social worker; (3) an interactive session on the caregiving experience, facilitated by a former caregiver; (4) an observed standardized patient scenario in the simulated apartment highlighting safety hazards in the home environment, commented on by an occupational therapist; and (5) a debrief with workshop participants.

**Results:** Since its creation as a pilot project in Fall 2017, our team has successfully hosted 6 workshops and engaged a total of 87 caregivers and 77 families, with 2 more workshops anticipated for Spring 2019. Ninety-five percent of participants have found the topics covered very relevant and 92% of participants reported an increased understanding of the disease following the workshops.

**Discussion:** To our knowledge, this is the first simulation-based workshop available to dementia caregivers. As the project evolves, our goal is to offer these workshops to a larger demographic through online modules and distance blended learning. We are also making steps towards bringing this workshop to medical students in order to sensitize them to the challenges faced by dementia caregivers and better prepare them to work with dementia patients and their families.

